# Profound and Sustained Reduction in *Chlamydia trachomatis* in The Gambia: A Five-Year Longitudinal Study of Trachoma Endemic Communities

**DOI:** 10.1371/journal.pntd.0000835

**Published:** 2010-10-05

**Authors:** Matthew J. Burton, Martin J. Holland, Pateh Makalo, Esther A. N. Aryee, Ansumana Sillah, Sandra Cohuet, Angels Natividad, Neal D. E. Alexander, David C. W. Mabey, Robin L. Bailey

**Affiliations:** 1 London School of Hygiene and Tropical Medicine, London, United Kingdom; 2 Medical Research Council Laboratories, Fajara, The Gambia; 3 National Eye Care Programme, Banjul, The Gambia; 4 Institut de Veille Sanitaire, Saint-Maurice, France; Oswaldo Cruz Foundation, Brazil

## Abstract

**Background:**

The elimination of blinding trachoma focuses on controlling *Chlamydia trachomatis* infection through mass antibiotic treatment and measures to limit transmission. As the prevalence of disease declines, uncertainty increases over the most effective strategy for treatment. There are little long-term data on the effect of treatment on infection, especially in low prevalence settings, on which to base guidelines.

**Methodology/Principal Findings:**

The population of a cluster of 14 Gambian villages with endemic trachoma was examined on seven occasions over five years (baseline, 2, 6, 12, 17, 30 and 60 months). Mass antibiotic treatment was given at baseline only. All families had accessible clean water all year round. New latrines were installed in each household after 17 months. Conjunctival swab samples were collected and tested for *C. trachomatis* by PCR. Before treatment the village-level prevalence of follicular trachoma in 1 to 9 year olds (TF_%1–9_) was 15.4% and *C. trachomatis* was 9.7%. Antibiotic treatment coverage was 83% of the population. In 12 villages all baseline infection cleared and few sporadic cases were detected during the following five years. In the other two villages treatment was followed by increased infection at two months, which was associated with extensive contact with other untreated communities. The prevalence of infection subsequently dropped to 0% in these 2 villages and 0.6% for the whole population by the end of the study in the absence of any further antibiotic treatment. However, several villages had a TF_%1–9_ of >10%, the threshold for initiating or continuing mass antibiotic treatment, in the absence of any detectable *C. trachomatis*.

**Conclusions/Significance:**

A single round of mass antibiotic treatment may be sufficient in low prevalence settings to control *C. trachomatis* infection when combined with environmental conditions, which suppress transmission, such as a good water supply and sanitation.

## Author Summary

Trachoma is the most common infectious cause of blindness worldwide. Mass antibiotic treatment with azithromycin is used to control ocular *Chlamydia trachomatis* infection. There is uncertainty over how frequently and for how long treatment is needed, particularly in low prevalence settings. This study examines the effect of a single round of treatment on clinical disease and infection in a cluster of trachoma endemic Gambian villages over a five-year period. These villages had good water supplies and sanitation improved part way through the study. We found treatment was followed by a marked decline in infection prevalence (by PCR) to less than 1%. The decline in prevalence of active disease in children was less marked. Several villages had a prevalence of active trachoma in 1 to 9 year old children of greater than 10% during the follow-up period, mostly in the absence of detectable infection. The implication of this study is that a single, high coverage mass treatment may be sufficient to control *C. trachomatis* infection in a low prevalence setting, particularly when combined with environmental measures to limit transmission. However, relying on clinical signs to guide treatment decisions is likely to lead to significant amounts of over treatment where current guidelines are implemented.

## Introduction

Trachoma is the leading infectious cause of blindness worldwide [1]. Repeated infection of the ocular surface by *Chlamydia trachomatis*, provokes conjunctival inflammation (active trachoma) and scarring. This may progress in some people to entropion (in-turned eyelid), trichiasis (TT, lashes scratching the cornea) and blinding corneal opacification. The World Health Organization's (WHO) most recent estimates indicate approximately 40 million people have active trachoma, 8 million have un-operated trichiasis and 1.3 million are blind [1], [2]. The burden of this disease falls disproportionately on poor rural communities, predominantly in Sub-Saharan Africa. In a major WHO-led effort to control trachoma, the Alliance for the Global Elimination of Blinding Trachoma by 2020 (GET2020) was formed which focuses on the development and implementation of the SAFE Strategy: **S**urgery for trichiasis, **A**ntibiotics for infection, **F**acial cleanliness, and **E**nvironmental improvements to suppress transmission [3].

Trachoma was endemic in many parts of Europe in the 19^th^ century; however, it gradually declined in the absence of any trachoma-specific interventions such as antibiotic treatment. This was attributed to general improvements in living conditions and hygiene, and underlines their importance in controlling trachoma. Unfortunately, such socio-economic transformation is only arriving slowly in many of today's trachoma endemic regions. Through implementing control programmes some encouraging reductions are being achieved. The Gambia, for example, has seen a marked decline [4]. Outside Sub-Saharan Africa, Morocco, Iran, Oman and Mexico have reported elimination of blinding trachoma. However, other countries have yet to experience similar reductions, probably because of higher initial levels of disease and variations in environmental factors such as water availability [2].

Typically, control programmes rely on the clinical signs of active trachoma to determine whether treatment should be given or stopped. Unfortunately, there is often a mismatch between disease and infection: infection may detected without clinical disease and disease may be found without infection. The correlation between disease and infection is not particularly strong and tends to weaken as the community prevalence drops [5], [6]. This led the WHO to recommend the use of mass drug administration (MDA) with either single –dose oral azithromycin (which has therapeutic tissue concentrations for several days) or topical tetracycline to entire communities or districts [7]. Revised recommendations were published in 2004 with emphasis placed on the prevalence of follicular trachoma in children aged 1–9 years (TF_%1–9_) as the primary clinical indicator [8]. Where TF_%1–9_ exceeds 10%, three annual MDAs followed by re-assessment, to determine whether to continue treating, is recommended [8]. This strategy increases the chance that infected persons are treated. However, in low prevalence settings where infection and disease are poorly correlated it may not represent the most efficient way to eliminate trachoma. The WHO also published specific Ultimate Intervention Goals (UIG); targets that need to be reached for the national certification of elimination of blinding trachoma [8]. For the surgery component of the SAFE strategy the UIG is to bring the population prevalence of TT below 0.1% in people aged 15 years and over. For the “AFE” components the UIG is to bring TF_%1–9_ below 5%. At this level it is expected that *C. trachomatis* infection will be too infrequent to drive scarring, although, there are no published data demonstrating this.

The efficient and timely elimination of blinding trachoma is a major challenge for prevention of blindness programmes. It presents various practical difficulties, which need to be overcome if, for example, regular high coverage with antibiotic treatment is to be achieved. In addition, there are significant areas where the evidence base which guides policy, particularly around the use of antibiotic treatment, needs to be strengthened. In particular, there are limited published long-term data from low prevalence settings on *C. trachomatis* infection and disease to inform policy makers and programme managers in the development of guidelines for handling the end stage of trachoma elimination. Several key questions need to be answered in a range of endemic settings. Who should be treated? How often should treatment be given? What indicators can be used to guide the treatment decision? When can treatment be discontinued? What form of surveillance is needed after discontinuing treatment? What environmental changes reinforce the reduction achieved by antibiotic?

We investigated the long-term effect of a single MDA on both the signs of trachoma and ocular *C. trachomatis* infection in a low prevalence setting in The Gambia. Other components of the SAFE strategy were also implemented, without any additional antibiotic treatment. Here we present results to five years; the first 17 months have previously been reported [9].

## Methods

### Ethical permission

Approval for this study was given by the Gambian Government/Medical Research Council Joint Ethics Committee (SCC Number: 856) and by the London School of Hygiene & Tropical Medicine Ethics Committee. Informed consent at village, family and individual level was required before enrolment.

### Study area and population

A rapid assessment of active trachoma in children was conducted in 31 villages in Upper and Lower Saloum Districts, The Gambia. A geographically defined study area was selected, which contained a group of communities with endemic trachoma. This cluster of villages was chosen as it was typical of rural Gambia, where there is marked variation in the prevalence of active disease between neighbouring communities. The study area population was enumerated and information was collected on latrine access, water supply, livestock, house construction and other socio-economic indicators. Individuals normally resident in the study area for at least 6 months of the year were enrolled. Visitors were excluded. New residents were included in the study population if they lived in the study area for at least six months. For the first 17 months of this study each household was visited weekly by a project fieldworker to update the census. Inward and outward travel was recorded. Episodes of illness and the treatment, especially antibiotics, were also recorded. The weekly visits stopped after the first 17 months. The census was updated again just prior to the 30 and 60-months follow-ups. At five years household access to a functional pit latrine was reassessed.

### Clinical assessment

The study population was assessed on seven occasions over five years: baseline (April 2001), 2, 6, 12, 17, 30 and 60 months. The previously reported 6 and 12 month data are not presented here [9]. On each occasion all individuals normally resident within the study area were eligible for assessment. The assessments were conducted within a 6 to 10 day period. Individuals who had travelled out of the study area for this period were not seen. All clinical observations were made by the same ophthalmologist. Faces were assessed for fly-eye contact and the presence of nasal and ocular discharge. The left eye of each subject was examined for trachoma and classified using the WHO Trachoma Grading System [10]. Clinically active trachoma was defined as the presence of either a follicular score of 2 or 3 (F2/3) or a papillary score of 3 (P3), equivalent to Trachomatous Inflammation, Follicular (TF) and Trachomatous Inflammation, Intense (TI) of the WHO Simplified Trachoma Grading System, respectively [11]. A Dacron swab sample was collected from the left upper tarsal conjunctiva, in a standardised manner, for the detection of *C. trachomatis* [9]. Care was taken to minimise the risk of contamination, by carefully handling only the far end of the swab (away from the Dacron head) and washing of the examiner's hands between subjects. All samples were kept on ice packs until transfer to a −20°C freezer later the same day for storage until processing.

### Antibiotic treatment

Mass antibiotic treatment of the entire population of the study area was conducted by the study team immediately after the baseline clinical assessment in April 2001. Children (<16 years) were offered a single oral dose of Azithromycin (suspension, 20mg/kg, up to a maximum of 1g). Infants under six months were given tetracycline eye ointment (1%, twice daily for six weeks). Adults were treated with a single oral dose of Azithromycin (tablets, 1g), except for women of childbearing age who received oral erythromycin, as per the Gambian National Treatment Guidelines at that time (500mg, twice daily for two weeks). During the follow-up period no further treatment was given for active trachoma by the study team or the National Eye Care Programme (NECP). The final five year follow-up took place after the introduction of the current WHO trachoma treatment guidelines, therefore, at the end of the study all villages with a TF_%1–9_ of 10% or more received mass antibiotic treatment as outlined above [8].

### Other trachoma control measures

All individuals with trachomatous trichiasis were offered surgery, which was provided free of charge within the community. After the 17-month follow-up new concrete pit latrines were installed in each household. The project team provided health education about trachoma to the communities involved throughout the course of the study.

### Detection of *C. trachomatis*

*C. trachomatis* was detected using the Amplicor CT/NG kit (Roche Molecular Systems, Branchburg, NJ). For samples in which *C. trachomatis* was detected by Amplicor, the infection load was estimated by quantitative real-time PCR of the *C. trachomatis ompA* gene using a previously described methodology [12]. The quantitative PCR method used was based on the *ompA* gene sequence of C. *trachomatis* serovar A (Forward Primer: 5′-GCTGTGGTTGAGCTTTATACAGACAC-3′, Reverse Primer: 5′-TTTAGGTTTAGATTGAGCATATTGGA-3′). For the 30 and 60 month *ompA* quantitation an additional forward primer (5′-TCTGTTGTTGAGTTGTATACAGATAC-3′) was used which was optimised for estimating serovar B. Quantitation was done on two 4 µL replicate samples, first with the serovar A forward primer and then with that for serovar B.

### Data analysis

Here we focus on the prevalence of TF and *C. trachomatis* infection in 1 to 9 year olds due to the importance placed on this group in the WHO guidelines [8]. Because of the duration of this study, many of those who were initially in the 1 to 9 year old group were above this range by the end. As the probability of being infected is strongly influenced by age, it is necessary to compare the same age group rather than the same individuals over the duration of the study. Hence data were analysed according to each person's contemporaneous age at the different study time points; e.g. we compared all children aged 1 to 9 years at baseline with all those who were aged 1 to 9 years at five years. The estimated number of copies of *ompA*/swab was the geometric mean of two replicate assays. If infection was detected by Amplicor but no *ompA* could be detected by quantitative PCR, a maximum likelihood copy number estimate was made [12]. Where no *C. trachomatis* was detected a copy number of zero was assigned. The estimated copy numbers in individual samples were combined into an estimate of the burden of chlamydia infection for the study population as a whole [13]. An adjusted geometric mean (the Williams Mean) was calculated by adding 1 copy/swab to each estimated *ompA* copy number, calculating the geometric mean and then subtracting 1 copy/swab from the result. This measure can be used where one or more zero data values make the true geometric mean zero. To compare the prevalence of TF_%1–9_ and infection at the final five year follow-up with those at baseline, the within-village differences in prevalence were bootstrapped with 100,000 replicates [14]. For this last analysis, villages were given equal weight irrespective of numbers sampled. However, results based on minimum variance weights, taking into account both numbers sampled and intra-village correlation, were identical at the number of decimals places used [15]. Data were analysed in STATA version 10 and R version 2.

## Results

### Study population

The study population was comprised of all the individuals living in a geographically defined area 3km by 4km in size in Upper Saloum District, The Gambia. This area contains a cluster of 14 small villages, which have previously been described in detail [6], [9]. At baseline in April 2001 the study area had a total recorded population of 1595 people. The median age was 14.2 years (Interquartile range: 6.2–30.2 years) and there were 542 children aged 1 to 9 years. The mean village size at baseline was 115 people; the individual village population sizes are shown in Table 1. One village (No. 12) of 123 people withdrew from the study after the 17-month follow-up. During the course of the five years we recorded 400 births, 57 deaths, 405 new residents, and 567 people moving away. At five years the total population of the study area (excluding village 12) had risen slightly to 1667 people. The age structure was comparable: median age 13.5 years (Interquartile range 5.5–30.5 years).

**Table 1:** Baseline village populations and antibiotic treatment coverage.

Village	All ages			Children 1–9 years		
	Total	Treated	(%)	Total	Treated	(%)
1	167	143	(85.6)	58	50	(86.2)
2	109	98	(89.9)	41	37	(90.2)
3	128	118	(92.2)	48	45	(93.8)
4	316	258	(81.7)	124	114	(91.9)
5	142	107	(75.4)	54	47	(87.0)
6	61	40	(65.6)	15	13	(86.7)
7	97	87	(89.7)	31	31	(100.0)
8	99	83	(83.4)	22	22	(100.0)
9	26	23	(88.5)	5	5	(100.0)
10	70	59	(84.3)	22	21	(95.5)
11	119	98	(82.4)	38	34	(89.5)
12	123	103	(83.7)	39	35	(89.7)
13	68	52	(76.5)	24	21	(87.5)
14	70	59	(84.3)	21	20	(95.2)
Study Area	1595	1328	(83.2)	542	495	(91.3)

The villages in the study area are typical of rural Gambia: the family compounds are grouped closely together and surrounded by farmland. The main employment is subsistence farming. All the households in the study area had access to clean water all year round within a few minutes' walk from wells situated in the middle of each village. At baseline 683/1595 (42.8%; 95% CI: 40.4–45.2) of the study population had access to a functional latrine in the family compound, although this varied considerably from village to village (Table 2). We have previously reported that at baseline there was a strong association between *C. trachomatis* infection and not having access to a latrine (OR 13.3, p<0.0001) [9]. Therefore, shortly after the 17-month follow-up visit, new pit latrines were installed in all 114 households. At five years 1154/1667 (69.2%; 95% CI: 67.0–71.4) of the population had access to a functional latrine (Table 2).

**Table 2:** Latrine access at baseline and 5 year, by village (all ages).

Village	All ages – Baseline			All Ages – 60 Months		
	Total	Latrine Access	(%)	Total	Latrine Access	(%)
1	167	0	(0.0)	213	213	(100.0)
2	109	74	(67.9)	123	123	(100.0)
3	128	12	(9.3)	155	61	(39.3)
4	316	162	(51.3)	358	274	(76.5)
5	142	110	(77.5)	196	88	(44.9)
6	61	0	(0.0)	39	18	(46.2)
7	97	82	(84.5)	107	107	(100.0)
8	99	60	(60.6)	88	64	(72.7)
9	26	26	(100.0)	43	26	(60.5)
10	70	0	(0.0)	112	24	(21.4)
11	119	58	(48.7)	109	94	(86.2)
12	123	77	(62.6)	-	-	-
13	68	18	(26.5)	62	0	(0.0)
14	70	4	(5.7)	62	62	(100.0)
Study Area	1595	683	(42.8)	1667	1154	(69.2)

### Antibiotic treatment coverage

These communities had not received any mass antibiotic treatment for trachoma control prior to this study. Antibiotic treatment coverage at baseline was 83.3% (95% CI: 81.4–85.1) of the study area population and 91.3% (95% CI: 89.0–93.7) of 1 to 9 year olds: oral azithromycin 1079 (81%), oral erythromycin 226 (17%), topical tetracycline 23 (2%). Coverage by village is shown in Table 1 for all ages and those aged 1 to 9 years at baseline.

### Follow-up assessments

The follow-up rates of the 1 to 9 year old children were around 90%; individual village rates at each visit are shown in Table 3. During the first 17 months of the study 153 episodes of illness were recorded. Forty-five of these episodes involved treatment with systemic antibiotics, which were usually obtained from the district health centre 10km away. This was equivalent to 20 antibiotic treatments/1000 people/year, during that 17 month period. Co-trimoxazole (Septrin) was the commonest antibiotic used (61%) and tetracyclines the next commonest (16%).

**Table 3:** Follow-up rates for children aged 1 to 9 years at each time point, by village.

Village	2 months		6 months		12 months		17 months		30 months		60 months	
	%	(seen/total)	%	(seen/total)	%	(seen/total)	%	(seen/total)	%	(seen/total)	%	(seen/total)
1	80.4	(45/56)	88.7	(55/62)	86.7	(52/60)	81.4	(48/59)	92.5	(49/53)	93.5	(72/77)
2	100.0	(40/40)	97.4	(38/39)	91.7	(44/48)	88.4	(38/43)	90.0	(36/40)	90.2	(37/41)
3	83.0	(39/47)	95.7	(44/46)	84.1	(37/44)	86.1	(37/43)	92.0	(46/50)	85.3	(52/61)
4	94.3	(116/123)	90.8	(108/119)	69.0	(87/126)	92.6	(112/121)	91.7	(99/108)	89.2	(115/129)
5	88.9	(48/54)	88.9	(48/54)	85.2	(46/54)	90.0	(45/50)	85.4	(41/48)	98.7	(78/79)
6	100.0	(14/14)	92.9	(13/14)	83.3	(10/12)	84.6	(11/13)	100.0	(13/13)	87.5	(14/16)
7	90.0	(27/30)	93.1	(27/29)	83.9	(26/31)	93.6	(29/31)	84.4	(27/32)	100.0	(40/40)
8	90.9	(20/22)	90.0	(18/20)	67.9	(19/28)	87.5	(21/24)	83.3	(20/24)	92.0	(23/25)
9	100.0	(5/5)	100.0	(5/5)	60.0	(3/5)	75.0	(3/4)	83.3	(5/6)	83.3	(15/18)
10	86.4	(19/22)	82.6	(19/23)	80.0	(20/25)	96.5	(28/29)	90.0	(27/30)	94.7	(36/38)
11	92.1	(35/38)	100.0	(39/39)	82.1	(32/39)	94.6	(35/37)	89.5	(34/38)	88.2	(30/34)
12*	92.1	(35/38)	91.9	(34/37)	83.3	(30/36)	85.3	(29/34)	-	-	-	-
13	91.7	(22/24)	95.5	(21/22)	66.7	(16/24)	83.3	(20/24)	85.7	(24/28)	83.3	(20/24)
14	100.0	(21/21)	90.5	(19/21)	90.0	(18/20)	100.0	(20/20)	81.8	(18/22)	83.3	(10/12)
Study Area	91.0	(486/534)	92.1	(488/530)	79.7	(440/552)	89.5	(476/532)	89.2	(439/492)	91.3	(542/594)

*Village 12 withdrew after 17 months.

### Clinical findings

**Figure 1:**
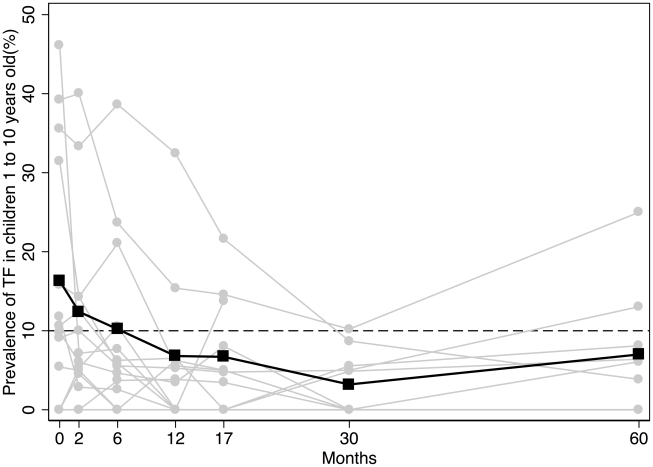
The prevalence of TF in children aged 1 to 9 years for the 14 villages*. Individual villages are represented by the grey lines. Several villages overlie each other with a prevalence of 0%. The overall village level average is represented by the black line. The dashed line represents the WHO TF_%1–9_ treatment initiation threshold level of 10%. *Village No. 12 withdrew after 17-months. TF = Trachomatous Inflammation, Follicular.

At baseline, the village-level average TF_%1–9_ was 15.4% (Table 4). There were marked differences in TF_%1–9_ between villages, ranging from 0% (in four) to 46%. It was greater than 10% in seven villages (Table 4). Overall, TF_%1–9_ declined during the study: reaching a minimum of 2.6% at 30 months and rising to 4.8% at five years (Figure 1). Comparing the baseline with five years, the village-level TF_%1–9_ decreased (in absolute terms, Table 4) by an average of 9% (95% CI −18 to −2, p = 0.002). Most villages had a reduction in this indicator within the first few months following MDA (Table 4). In contrast, in villages 1 and 3 it took longer to decline. By 30 months all villages had 10% TF_%1–9_ or less. At five years, six villages had cases of TF; two villages had a TF_%1–9_ of greater than 10% and were therefore retreated (Table 4). There was a marked reduction in observed fly-eye contacts at the time of examination in 1 to 9 year olds from 18.0% (95% CI 14.7 to 21.5) at baseline to 1.5% (95% CI 0.5–2.5) at 5 years. Similarly there was a large reduction in the proportion of 1 to 9 year olds with unclean faces (nasal or ocular discharge present): 38.4% (95% CI 34.1 to 42.7) at baseline and 5.0% (95% CI 3.1 to 6.8) at 5 years.

**Table 4:** Prevalence of follicular trachoma (TF) in children aged 1 to 9 years by village.

Village	Baseline		2 months		6 months		12 months		17 months		30 months		60 months	
	%		%		%		%		%		%		%	
1	39.2	(20/51)	40.0	(18/45)	23.6	(13/55)	15.4	(8/52)	14.6	(7/48)	10.2	(5/49)	25.0	(18/72)
2	5.4	(2/37)	5.0	(2/40)	0.0	(0/38)	0.0	(0/44)	0.0	(0/38)	5.6	(2/36)	8.1	(3/37)
3	35.6	(16/45)	33.3	(13/39)	38.6	(17/44)	32.4	(12/37)	21.6	(8/37)	8.7	(4/46)	3.9	(2/52)
4	9.8	(11/112)	6.0	(7/116)	4.6	(5/108)	3.4	(3/87)	8.0	(9/112)	0.0	(0/99)	6.1	(7/115)
5	10.6	(5/47)	12.5	(6/48)	6.3	(3/48)	6.5	(3/46)	0.0	(0/45)	4.9	(2/41	6.4	(5/78)
6	46.2	(6/13)	7.1	(1/14)	7.7	(1/13)	0.0	(0/10)	0.0	(0/11)	0.0	(0/13)	0.0	(0/14)
7	0.0	(0/31)	0.0	(0/27)	3.7	(1/27)	3.8	(1/26)	3.4	(1/29)	0.0	(0/27)	0.0	(0/40)
8	9.1	(2/22)	10.0	(2/20)	5.6	(1/18)	5.3	(1/19)	4.8	(1/21)	5.0	(1/20)	13.0	(3/23)
9	0.0	(0/5)	0.0	(0/5)	0.0	(0/5)	0.0	(0/3)	0.0	(0/3)	0.0	(0/5)	0.0	(0/15)
10	0.0	(0/21)	5.3	(1/19)	10.5	(2/19)	0.0	(0/20)	0.0	(0/28)	0.0	(0/27)	0.0	(0/36)
11	11.8	(4/34)	2.9	(1/35)	2.6	(1/39)	0.0	(0/32)	0.0	(0/35)	0.0	(0/34)	0.0	(0/30)
12*	31.4	(11/35)	14.3	(5/35)	5.9	(2/34)	0.0	(0/30)	13.8	(4/29)	-	-	-	-
13	0.0	(0/20)	4.5	(1/22)	0.0	(0/21)	6.3	(1/16)	5.0	(1/20)	0.0	(0/24)	0.0	(0/20)
14	15.8	(3/19)	14.3	(3/21)	21.1	(4/19)	5.6	(1/18)	5.0	(1/20)	0.0	(0/18)	0.0	(0/10)
**Village Level**														
Mean (95% CI)	15.4	(6.1–24.6)	11.1	(4.3–17.9)	9.2	(2.9–15.7)	5.6	(3.9–13.7)	5.4	(1.5–9.4)	2.6	(0.4–4.9)	4.8	(0.4–9.3)

*Village 12 withdrew after 17 months.

Trichiasis (TT) was found in 9/592 (1.5%) people 15 years and over at baseline. Six of these individuals accepted surgery by 12 months. At five years TT was found in 6/456 (1.3%) aged 15 years and over. Two were incident cases (compared with baseline), three had TT at baseline (one of which had received surgery) and one was a new resident at five years.

### *C. trachomatis* infection

Before treatment, the village-level average prevalence of *C. trachomatis* infection in 1 to 9 year olds was 9.7% (Table 5). There was marked variation in prevalence between the study villages despite their close proximity, with 8 villages having none. There were 7 villages where the TF_%1–9_ was greater than 10%, but only 4 of these had any cases of chlamydial infection.

**Table 5:** Prevalence of *C.trachomatis* infection* in children aged 1 to 9 years by village.

Village	Baseline		2 months		6 months		12 months		17 months		30 months		60 months	
	%		%		%		%		%		%		%	
1	5.9	(3/51)	20.0	(9/45)	20.0	(11/55)	11.5	(6/52)	16.7	(8/48)	4.1	(2/49)	0.0	(0/72)
2	0.0	(0/37)	2.5	(1/40)	2.6	(1/38)	9.1	(4/44)	2.6	(1/38)	5.6	(2/36)	0.0	(0/37)
3	53.3	(24/45)	59.0	(23/39)	25.0	(11/44)	35.1	(13/37)	29.7	(11/37)	0.0	(0/46)	0.0	(0/52)
4	5.4	(6/112)	0.0	(0/116)	0.0	(0/108)	0.0	(0/87)	0.0	(0/112)	3.0	(3/99)	1.7	(2/115)
5	0.0	(0/47)	0.0	(0/48)	0.0	(0/48)	0.0	(0/46)	0.0	(0/45)	0.0	(0/41	1.3	(1/78)
6	15.4	(2/13)	0.0	(0/14)	0.0	(0/13)	0.0	(0/10)	0.0	(0/11)	0.0	(0/13)	0.0	(0/14)
7	3.2	(1/31)	0.0	(0/27)	0.0	(0/27)	0.0	(0/26)	0.0	(0/29)	0.0	(0/27)	0.0	(0/40)
8	0.0	(0/22)	0.0	(0/20)	0.0	(0/18)	0.0	(0/19)	0.0	(0/21)	0.0	(0/20)	0.0	(0/23)
9	0.0	(0/5)	0.0	(0/5)	0.0	(0/5)	0.0	(0/3)	0.0	(0/3)	0.0	(0/5)	0.0	(0/15)
10	0.0	(0/21)	0.0	(0/19)	0.0	(0/19)	0.0	(0/20)	0.0	(0/28)	0.0	(0/27)	0.0	(0/36)
11	0.0	(0/34)	0.0	(0/35)	0.0	(0/39)	0.0	(0/32)	0.0	(0/35)	0.0	(0/34)	0.0	(0/30)
12†	0.0	(0/35)	0.0	(0/35)	0.0	(0/34)	3.3	(1/30)	0.0	(0/29)	-	-	-	-
13	0.0	(0/20)	0.0	(0/22)	4.8	(1/21)	0.0	(0/16)	0.0	(0/20)	0.0	(0/24)	0.0	(0/20)
14	52.6	(10/19)	0.0	(0/21)	0.0	(0/19)	0.0	(0/18)	0.0	(0/20)	0.0	(0/18)	0.0	(0/10)
**Village Level**														
Mean (95% CI)	9.7	(0.0–20.6)	5.8	(0.0–15.2)	3.7	(0.0–8.4)	4.2	(0.0–9.8)	3.5	(0.0–8.6)	1.0	(0.0–2.1)	0.2	(0.0–0.6)

*C. trachomatis infection detected by Amplicor.

**†:** Village 12 withdrew after 17 months.

**Figure 2:**
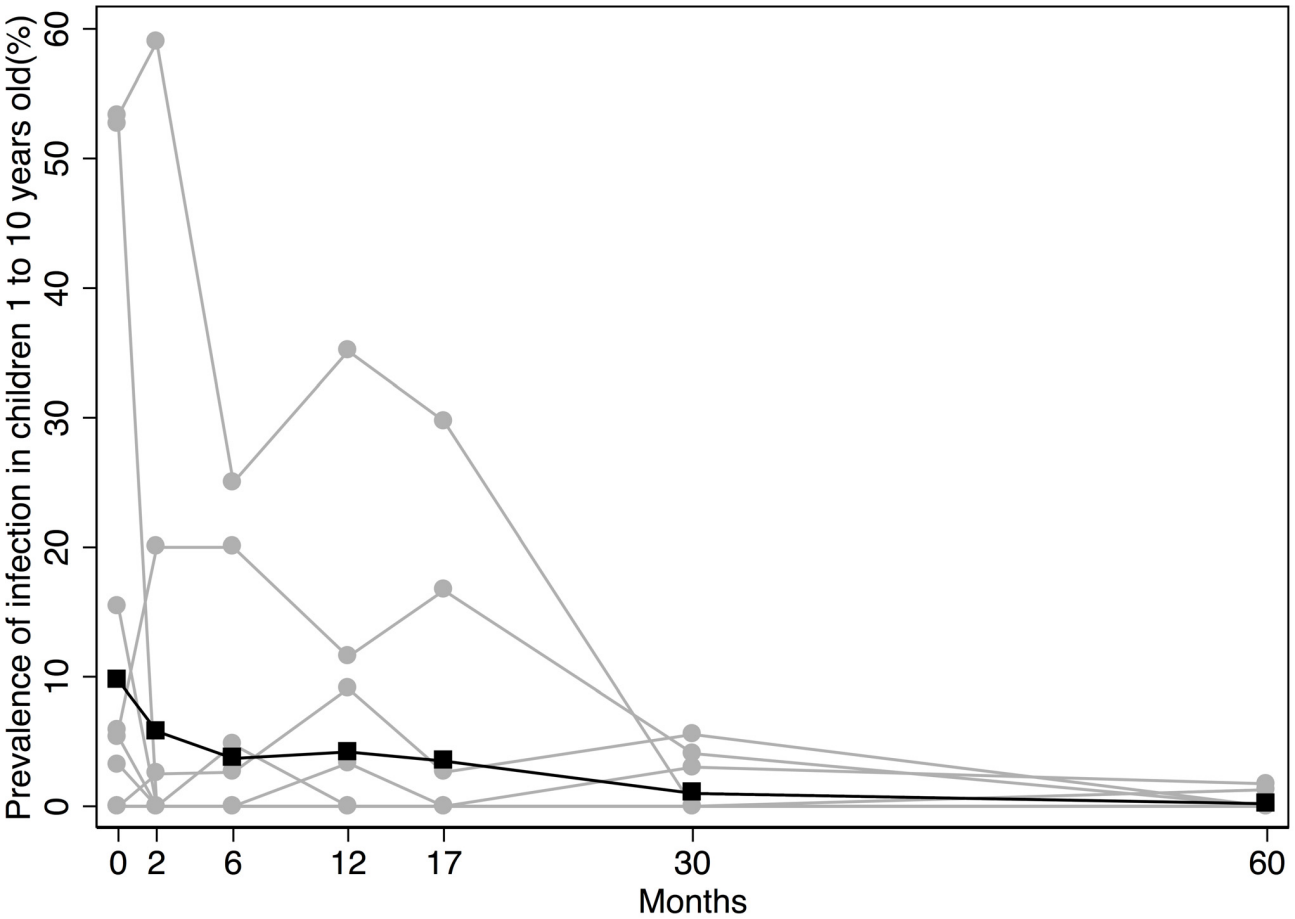
The prevalence of *Chlamydia trachomatis* infection* in children aged 1 to 9 years for the 14 villages^†^. Individual villages are represented by the grey lines. Several villages overlie each other with a prevalence of 0%. The overall village level average is represented by the black line. *Chlamydia trachomatis infection determined by Amplicor CT PCR assay. ^†^Village No. 12 withdrew after 17-months.

Following MDA the prevalence of *C. trachomatis* infection declined gradually, dropping below 1% by five years (Figure 2 & Table 5). Comparing the baseline with five years, the village-level prevalence of *C. trachomatis* infection (in absolute terms, Table 5) decreased by 10% (95% CI −22 to −1, p = 0.001). We have previously reported the village level heterogeneity in the initial response to treatment [9]. In most villages (12) all cases of infection identified before treatment had cleared by two months (Table 5), and over the subsequent five years we detected only 12 isolated cases of infection in those villages. In contrast, in villages 1 and 3 the prevalence of infection increased at two months (Table 5). This increase was strongly associated with the travel of most village residents to a religious festival in Senegal one month after receiving treatment, which has been previously reported (OR 12.2, p<0.0001, 95% CI 4.0–44.1) [9]. After the 2-month assessment, infection prevalence in these two villages declined progressively to zero at five years (Table 5). The risk of infection at any follow-up time point did not differ between those who had received treatment at baseline and those who had not or who were new residents during the follow-up (data not shown).

After the two-month follow-up TF_%1–9_ was found to exceed the 10% treatment threshold in several villages on one or more occasions (Table 4). However, in only 4/12 (33%) of these occasions were cases of chlamydial infection detected in the same village (Table 5). Conversely, after the two-month follow-up chlamydial infection was detected in samples from several villages on one or more occasions (Table 5). However, TF_%1–9_ exceeded 10% on only 6/16 (37.5%) of these occasions (Table 4).

The geometric mean load of *C. trachomatis* infection amongst infected individuals (all ages) increased after treatment (Table 6). This burden of infection was concentrated in villages 1 and 3. At 30 and 60 months we found very few infected individuals, most of whom had low infection loads. The estimated community *C. trachomatis* burden (Williams mean) declined throughout the five-year follow-up period (Table 6).

**Table 6:** *Chlamydia trachomatis* infection load.

	Baseline		2 months		6 months		12 months		17 months		30 months		60 months	
	Load	(95%CI)	Load	(95%CI)	Load	(95%CI)	Load	(95%CI)	Load	(95%CI)	Load	(95%CI)	Load	(95%CI)
Individual*	169	(100–286)	186	(103–333)	879	(371–2083)	620	(229–1681)	400	(92–1742)	72	(14–371)	17	(5–59)
Community†	0.44	(0.33–0.57)	0.34	(0.24–0.44)	0.24	(0.16–0.33)	0.20	(0.12–0.28)	0.14	(0.08–0.21)	0.07	(0.02–0.09)	0.02	(0.01–0.04)

*The individual infection load is the geometric mean and 95% CI of the *C. trachomatis* infection load expressed in copies of *ompA*/swab, for all infected individuals in the study population.

**†:** The community load of infection is the Williams Mean for the study population (including uninfected individuals), expressed in copies of the *ompA*/swab.

## Discussion

In this study a single round of mass antibiotic treatment was given to a cluster of 14 Gambian villages with low prevalence trachoma (TF_%1–9_ 15.4%). In addition, the other components of the SAFE strategy were promoted: free surgery was offered for trichiasis, some limited health education on trachoma was provided and latrines were installed at 18 months. The government had previously improved the water supply. Prior to treatment there were marked variations in village-level prevalence of disease and infection, despite their close proximity and environmental similarity. Six villages had cases of infection before treatment. The initial response to treatment was heterogeneous. In four villages with pre-treatment infections, no infection was detected at 2-months, seven villages had no infection at baseline or at 2-months and one village which had no baseline infection had a single new case at 2-months. In these 12 villages only a few isolated cases were subsequently detected during the five years of follow-up. In the other two villages there appeared to be a failure to respond to treatment [9]. This was attributed to possible re-infection, because it was associated with *en masse* travelling to a large religious festival in Senegal. Thereafter, the prevalence of infection in these two villages gradually declined in the absence of any additional antibiotic treatment for trachoma, reaching zero by five years. In the absence of additional antibiotic treatment, this decline is likely to be attributable to favourable environmental factors. It is interesting to note that there was less *Ct* strain diversity two-months after the mass treatment, which may have also contributed to the decline in infection [16]. Overall, these observations suggest that: (1) a single MDA was effective in most of the study area, although re-infection seemed to have occurred in two village; (2) the environmental conditions and/or the susceptibility of the population did not favour the transmission of *C. trachomatis*, leading to its decline and failure to re-emerge.

These findings contrast those from highly endemic regions such as Ethiopia where *C. trachomatis* infection is not easily controlled or readily re-emerges. For example, a study of a single MDA to eight Ethiopian villages documented an initially good response to treatment, but subsequently, several villages experienced re-emergent infection [17]. Even where MDA was given every six months for two years and the village prevalence of infection dropped to very low levels or zero, re-emergence of infection was reported two years after treatment stopped [18]. However, even in these highly endemic parts of Ethiopia local elimination may be possible with more prolonged treatment [19].

The Gambia is currently considered to have low levels of endemic trachoma, although individual districts have TF_%1–9_ above the threshold for MDA [20]. Historically the levels were somewhat higher, with a well-documented downward trend during recent decades [4], [21]. This reduction has been largely in the absence of a MDA programme. The Gambia became a beneficiary of the azithromycin donation programme through the International Trachoma Initiative in 2007. Prior to this, tetracycline eye ointment was used to opportunistically treat individuals with active disease and their families. Therefore, it seems more likely that the decline in the prevalence of trachoma is mainly due to other factors.

Various environmental factors may favour this change. However, it is difficult to determine their relative contributions. All households in the study area had access to clean water within a few minutes' walk. Access to water improved markedly throughout The Gambia during the 1990's through the provision of covered wells with hand-pumps by the government and coincided with a general reduction in trachoma [4]. A similar transition was observed in Malawi, which also coincided with marked improvements in water supply and hygiene programme [22]. Easier access to water combined with hygiene promotion probably leads to improved facial cleanliness and washing of bed linen and clothes. Numerous cross-sectional studies have found an association between active trachoma and unclean faces [23], [24]. This led to the hypothesis that improving facial cleanliness may help to control trachoma by suppressing transmission of *C. trachomatis* in ocular secretions. However, the causality in this relationship could go both ways, as active conjunctival inflammation can produce secretions. This question was assessed in a trial of promoting facial cleanliness, which found a reduction in severe trachoma (TI) but not “any trachoma” (TF and/or TI) [25]. In our study we observed a marked reduction in the prevalence of unclean faces as the prevalence of TF_%1–9_ declined. We do not know whether this promoted or was secondary to the decline in active trachoma or whether it was related to health education about facial cleanliness.

Eye seeking flies (principally *Musca sorbens*) are thought to contribute to the transmission of *C. trachomatis* in The Gambia. Measures to suppress the fly population by insecticide spraying have been associated with a drop in the prevalence of active trachoma [26], [27]. As this fly preferentially breeds in exposed human faeces, the construction and use of pit latrines may indirectly reduce the transmission of infection [28]. However, in a cluster randomised trial from the Gambia the provision of pit latrines was only associated with a non-significant reduction in the prevalence of active trachoma [27]. In our study latrine coverage improved from 43% at baseline to 69% at five years. At the same time fly-eye contacts dropped significantly. However, we do not know whether this was due to a reduction in the overall fly population secondary to the latrines or a reduction in unclean faces, which attract flies. Whatever the explanation, reducing the frequency of fly-eye contact probably reduces the probability that *C. trachomatis* (if present) will be transmitted by this route in this particular environment. In contrast, in a study from Tanzania, intensive insecticide spraying was not associated with a reduction in trachoma [29]. This may reflect differences in the transmission ecology of this infection in different regions.

The study population did not have ready access to antibiotics with anti-chlamydial activity. Antibiotic use was monitored during the first 17 months of the study, and we have no reason to think that it was different during the rest of the follow-up period. Background antibiotic use probably contributed little to the decline in *C. trachomatis*, in contrast to a study from Nepal, where a much higher background use of antibiotics may have contributed to a decline in trachoma [30].

During our study there were no significant demographic changes, such as fewer young children, favouring trachoma control. The relatively small village size in this part of The Gambia may make trachoma less stable. In a smaller community children probably come into contact with a smaller absolute number of potentially infected people than in a larger village, possibly reducing their chance of becoming infected.

A difficult issue facing trachoma control programmes as the prevalence of disease declines is the increasing uncertainty over the infection status of formerly endemic communities, because of a mismatch between disease signs and chlamydial infection [5]. We have previously reported that prior to MDA in these villages a minority of the people with *C. trachomatis* had TF and conversely many with clinically active trachoma did not have infection [6], [9]. At the five year follow-up two villages had a TF_%1–9_ of >10%, triggering intervention with MDA, under current WHO guidelines [8]. However, neither village had a single case of chlamydial infection. These observations are consistent with those from a survey conducted elsewhere in The Gambia in the same year (2006) as our five year follow-up [20]. With the increasing effectiveness of trachoma control programmes other countries are likely to reach the low prevalence situation current in The Gambia. The implication is that, applying the current intervention algorithms, many uninfected communities will continue to receive unnecessary rounds of MDA on multiple occasions. It remains uncertain, on clinical grounds, when MDA can stop without re-emergent infection.

The mismatch of disease and infection probably arises for several reasons in low prevalence settings. Firstly, there is a difference in the time course of episodes of *C. trachomatis* infection and active disease [31], [32]. There is probably an initial subclinical “incubation” period prior to the development of inflammatory signs in the conjunctiva; subsequently the inflammatory signs can linger for many weeks after the infection becomes undetectable. In contrast, in high prevalence settings this asynchronous time course is less evident, as the spacing between separate infection and disease episodes is shorter and may indeed overlap, increasing the correlation. Secondly, in low prevalence settings the disease is probably milder, with some infected individuals having conjunctival inflammatory signs, which do not reach the TF diagnostic threshold. Thirdly, follicular conjunctivitis is not exclusively caused by *C. trachomatis*. In low prevalence settings a greater proportion of “TF” will be due to other agents, such as adenovirus, causing outbreaks, which mimic endemic trachoma. In a number of studies the prevalence of TF after the successful control of *C. trachomatis* infection with MDA has taken several years to decline [9], [33], [34], [35]. The reason for this is not known, as most clinical episodes last only for a few weeks; however, it is likely that in many situations MDA will inevitably continue some time after the infection has been successfully cleared from formerly endemic communities. Finally, clinical observations and laboratory tests may both produce false negative and false positive results.

The observation that incident TT developed after the prevalence of *C. trachomatis* dropped is important. This indicates the potential for cicatricial disease progression after the chlamydial infection is controlled. The reasons for this are unknown, although other pro-inflammatory stimuli such as non-chlamydial bacterial infection and ocular dryness may contribute [36], [37]. The implication for control programmes is that surveillance and treatment for TT will need to continue for some years after the chlamydial infection has been successfully controlled.

This study has a number of limitations. Firstly, in the absence of an untreated control group, it is not possible to determine what proportion of the reduction seen in these villages was due to the treatment at baseline and what was due to a secular trend. However, it seems likely that both contributed to the decline. Secondly, there was a long gap between the last two time-points. It is unknown what occurred during that time. Thirdly, we do not routinely run parallel assays for human nucleic acid in swab samples to check specimen adequacy. However, in a separate study using the same swab type collected in the same standardised manner by the same ophthalmologist from subjects in this study area we found that human RNA could be extracted from all swabs collected [38]. Field control swabs were not collected, as this was not standard practice at that time. Finally, one village withdrew from the study after the first 17 months. At enrolment this village had one of the higher rates of TF_%1–9_ and on the last occasion it was assessed it had four cases of TF but no cases of infection. We do not know what happened in this village subsequently and what affect this might have had on the surrounding villages.

In summary, our findings suggest that a single round of mass antibiotic treatment in association with the other components of the SAFE strategy may be all that is required to clear *C. trachomatis* infection in low prevalence settings, if reasonably high antibiotic coverage is achieved. We also observed an unusual re-infection episode soon after treatment, which was associated with travel. However, the conditions were such that this infection did not persist or appear to transfer to neighbouring villages, indicating the critical importance of F&E interventions that suppress transmission, in addition to antibiotic treatment. Coordinated attempts to control trachoma across wide geographical areas may also reduce the risk of re-infection. The current guidelines will probably lead to widespread overtreatment of communities in similar settings. This might be preventable if a role for suitable field-based diagnostic tests for *C. trachomatis* can be established in confirming elimination of infection. With the implementation of the SAFE Strategy, many previously highly endemic regions will eventually reach this position. Therefore, a clear evidence-based approach for handling the trachoma “End Game” is still needed.
